# Engineered action at a distance: Blood-meal-inducible paralysis in *Aedes aegypti*

**DOI:** 10.1371/journal.pntd.0007579

**Published:** 2019-09-03

**Authors:** Roya Elaine Haghighat-Khah, Tim Harvey-Samuel, Sanjay Basu, Oliver StJohn, Sarah Scaife, Sebald Verkuijl, Erica Lovett, Luke Alphey

**Affiliations:** 1 Department of Life Sciences, Imperial College London, London, United Kingdom; 2 Arthropod Genetics Group, The Pirbright Institute, Woking, United Kingdom; 3 Department of Zoology, University of Oxford, Oxford, United Kingdom; 4 Immunocore, Park Drive, United Kingdom; University of California, Davis, UNITED STATES

## Abstract

**Background:**

Population suppression through mass-release of *Aedes aegypti* males carrying dominant-lethal transgenes has been demonstrated in the field. Where population dynamics show negative density-dependence, suppression can be enhanced if lethality occurs after the density-dependent (i.e. larval) stage. Existing molecular tools have limited current examples of such Genetic Pest Management (GPM) systems to achieving this through engineering ‘cell-autonomous effectors’ i.e. where the expressed deleterious protein is restricted to the cells in which it is expressed–usually under the control of the regulatory elements (e.g. promoter regions) used to build the system. This limits the flexibility of these technologies as regulatory regions with useful spatial, temporal or sex-specific expression patterns may only be employed if the cells they direct expression in are simultaneously sensitive to existing effectors, and also precludes the targeting of extracellular regions such as cell-surface receptors. Expanding the toolset to ‘non-cell autonomous’ effectors would significantly reduce these limitations.

**Methodology/Principal findings:**

We sought to engineer female-specific, late-acting lethality through employing the *Ae*. *aegypti* VitellogeninA1 promoter to drive blood-meal-inducible, fat-body specific expression of tTAV. Initial attempts using pro-apoptotic effectors gave no evident phenotype, potentially due to the lower sensitivity of terminally-differentiated fat-body cells to programmed-death signals. Subsequently, we dissociated the temporal and spatial expression of this system by engineering a novel synthetic effector (Scorpion neurotoxin–TetO-gp67.AaHIT) designed to be secreted out of the tissue in which it was expressed (fat-body) and then affect cells elsewhere (neuro-muscular junctions). This resulted in a striking, temporary-paralysis phenotype after blood-feeding.

**Conclusions/Significance:**

These results are significant in demonstrating for the first time an engineered ‘action at a distance’ phenotype in a non-model pest insect. The potential to dissociate temporal and spatial expression patterns of useful endogenous regulatory elements will extend to a variety of other pest insects and effectors.

## Author summary

A recent addition to the toolbox for controlling populations of the disease vector *Aedes aegypti* is the mass-release of males engineered with dominant, lethal transgenes. The lethal effect of these transgenes is activated in the progeny of these released engineered males and wild females they mate with in the field and with continuous release of males can cause population collapse. To date, these systems have relied on the use of ‘cell-autonomous’ effectors, meaning that their action is restricted to the cells in which they are expressed, limiting the flexibility of designing new, more complex systems. Here we demonstrate that it is possible to engineer ‘non-cell autonomous’ effectors–that is where the effect (e.g. the action of a toxic protein) can act on cells distant from the tissues in which they are originally expressed. To achieve this we utilised the endogenous cell secretory pathway to engineer a novel control phenotype–blood-meal inducible (i.e. late-acting, female-specific) reversible paralysis. The logic behind engineering such ‘action at a distance’ phenotypes will extend to a variety of other pest insects and control phenotypes.

## Introduction

Advances in molecular tools have allowed the development of a range of novel Genetic Pest Management (GPM) strategies [1, 2]. One such GPM strategy utilises the mass-release of males from the target species which have been genetically modified to express a repressible, dominant-lethal gene. In the wild, these released males mate wild females; in their offspring this lethality is not repressed so they die before reproducing. With successive releases, a target population can thus be reduced, potentially to the point of eradication [3–5]. Modelling has shown that, if the population dynamics of the target pest are regulated by negative density-dependence–reduced population growth at high population densities e.g. through competition for resources–suppression can be significantly enhanced if the engineered lethality occurs after the density-dependent phase, rather than before it as with radiation-sterilisation [4, 6]. System development for mosquitoes such as *Aedes aegypti* (the primary vector for dengue, Zika, chikungunya and yellow fever viruses) has therefore aimed to induce late-acting (e.g. pupae/adult) lethality as most density-dependent effects are evident at the larval stage. These ‘self-limiting’ GPM strategies have been extended to a wide variety of agricultural and human health pest insects, and have been successfully demonstrated in the field in multiple geographic locations [7–12].

**Fig 1:**
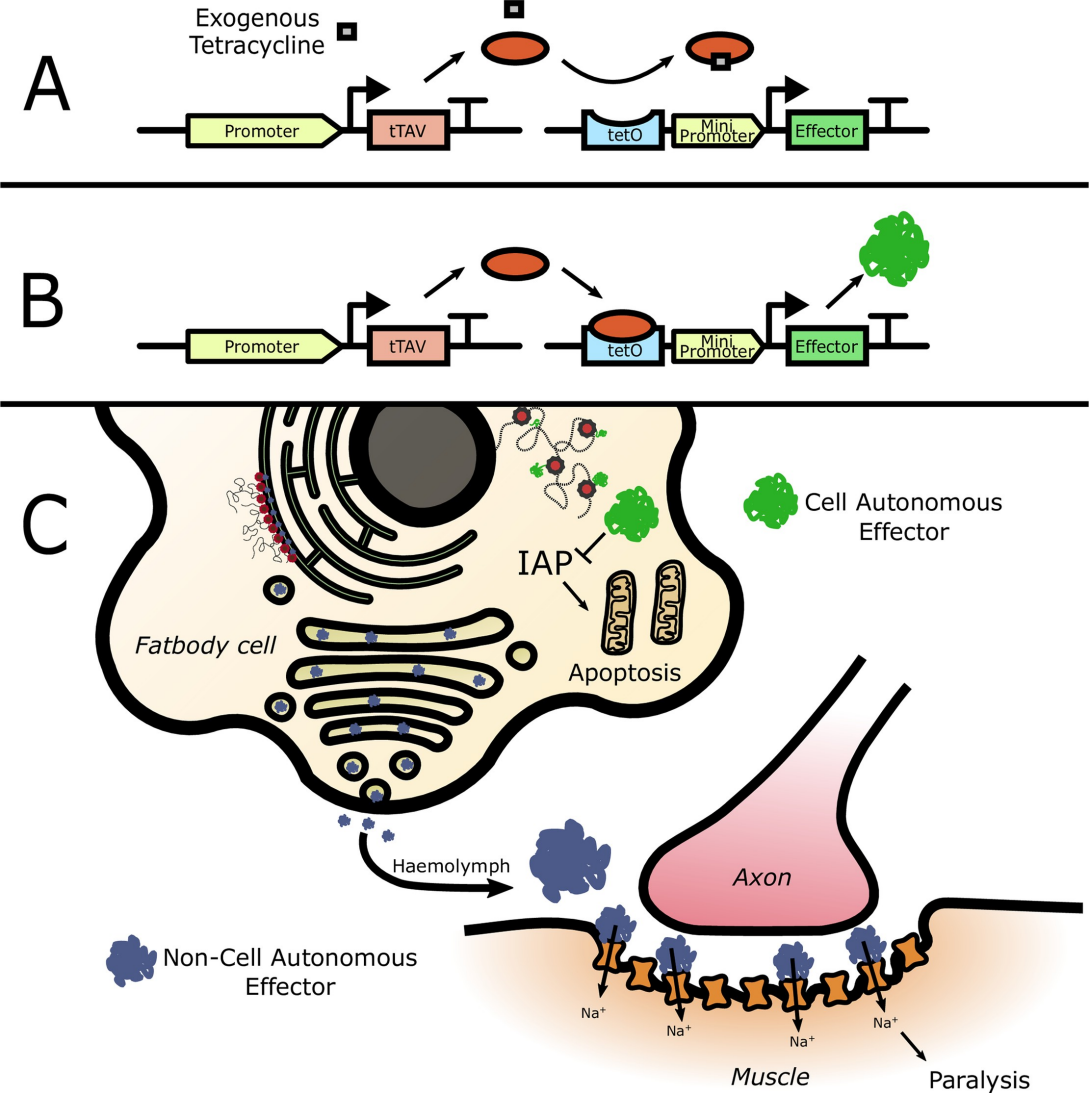
Schematic representation of the tet-off bipartite expression system and the use of cell-autonomous and non-cell autonomous effectors. A, B: The tet-off bipartite gene expression system. As used here, the system is divided into two genes carried by two independent transgenic lines. The ‘driver’ line will express the synthetic transcriptional activator tTAV under the spatial and temporal expression pattern of the promoter chosen to drive it (here from VitellogeninA1). The ‘effector’ line carries an effector open reading frame (here Michelob, Reaper^KR^ or AaHIT) under the transcriptional control of the tet operator (the binding site for tTAV protein). A: System in a suppressed state. Tetracycline (shown as a grey square) binds to the tTAV protein and prevents its binding to tetO, therefore, suppressing the expression of the downstream effector. B: System in an unsuppressed state. In the absence of tetracycline, tTAV will bind to tetO and stimulate expression of the effector. C: Comparison of the mode of action of cell autonomous and non-cell autonomous effectors. For the cell autonomous effector, mRNA leaving the nucleus is translated on ribosomes in the cytoplasm leading to protein which then sets off an intracellular pro-apoptotic cascade (as is the case for Reaper^KR^ and Michelob). For the non-cell autonomous effector secretory peptide signals (here gp67) lead it, instead, to be translated on ribosomes studding the endoplasmic reticulum (ER) and pass through the secretory pathway to the Golgi body where it is secreted into the haemolymph. In the case of AaHIT, this is made available to voltage-gated sodium channels at neuromuscular junctions leading to an influx of Na^+^ ions, cell depolarisation and muscle contraction (paralysis).

A variety of repressible lethal systems have been constructed in insects using the “tet-off” bipartite expression system [7, 13–16], allowing the engineered phenotype to be repressed prior to field release by providing transgenic animals with tetracycline or a suitable analogue (Fig 1A and 1B) [17]. Using this system, expression of an effector gene (tetO-effector) is controlled by the action of the tetracycline-repressible Transactivator (tTAV). In current designs, the spatial and temporal expression pattern of effector expression mirrors that of the regulatory elements used to drive tTAV. Whilst building systems in this manner has allowed novel control phenotypes e.g. sex-specific lethality/sterility [5, 16], it restricts the function of the system (i.e. the action of a toxic protein) to the cells in which tTAV is expressed. This current paradigm of using ‘cell-autonomous effectors’ is a significant limitation on developing more complex and flexible GPM technologies as it necessitates that the cell-type expressing the effector must simultaneously be sensitive to its effects, precluding the use of either a useful effector, or regulatory element, where this overlap does not occur. The potential to dissociate the temporal and spatial expression patterns of an effector (i.e. a ‘non cell-autonomous’ effector) would allow the use of a far wider panel of endogenous regulatory components for building GPM systems but, to date, has been limited by the available molecular tools.

We hypothesised that a route towards achieving non cell-autonomous effectors would be to use the secretory pathway of those cells engineered to show transcriptional activity as a mechanism for allowing effector proteins to dissociate away from these areas and affect distant tissues. Here, we demonstrate the successful use of such a system by engineering a novel synthetic effector designed to be secreted out of the adult fat-body following a blood-meal (a female-specific behaviour) and affect the functioning of motor neurons–leading to paralysis.

## Results and discussion

*Aedes aegypti* VitellogeninA1 (VgA1) is expressed primarily in the female fat body, following a blood meal [18–22]. Replicating this adult female-specific expression profile to drive a synthetic transactivator (tTAV) was the first integral step towards developing bloodmeal-inducible lethality using the bi-partite tet-off expression system (Fig 1A and 1B). We generated a transgenic line carrying tTAV driven by a VgA1-derived promoter sequence (VgA1-tTAV). Consistent with previous studies on VgA1, qPCR of a transgenic line carrying the VgA1-tTAV construct showed significant blood-meal-inducible upregulation of tTAV (mean = 182 x male pupae expression: *P* = 0.03, t = -5.56, df = 2) with very low basal expression in adult males and non-blood-fed females (S1 Fig). This induction occurred rapidly ca 24 h post blood-meal (pbm). Crossing this line to an existing tetO-DsRed2 reporter line and analysing transhemizygous progeny confirmed DsRed expression in the female fat body with some low level expression in males (S2 Fig), consistent with the tTAV qPCR data and previous transgenic characterisation of the VgA1 promoter fragment [18].

Initial attempts to pair this expression profile with a reduced-fitness phenotype–e.g. lethality–were unsuccessful. Crosses of the VgA1-tTAV line to tetO-Michelob_X and tetO-reaper^KR^ lines gave no discernible phenotype in transhemizygous females pbm, despite the previously established upregulation of the tTAV transactivator during this period and confirmed overexpression of the Michelob_x transcript in this context (S3 Fig)–overexpression behaviour of reaper^KR^ was assumed to follow a similar pattern to Michelob but was not directly assessed. Survival of double hemizygotes was not significantly different from controls (Kaplan-Meier survival analysis; Michelob: P = 0.616, X^2^ = 3.5, df = 5, Reaper^KR^: P = 0.677, X^2^ = 3.2, df = 5). These results were surprising as ectopic/exogenous expression of pro-apoptotic genes including Reaper and Michelob_x has previously been shown to be lethal in insect cell lines as well as causing tissue ablation *in vivo* [23–25]. Additionally, this tetO-Michelob_X effector line has previously been successfully used to cause disruption of female indirect flight muscles (Actin4 promoter) and hence flightlessness in *Ae*. *aegypti* [16]. One possible reason for this discrepancy may be due to an interplay between the differentiation stage of the cells being targeted in this study and previous studies and their relative sensitivities to programmed cell death signals. Whereas the expression profile of the Actin4 promoter used previously coincides with the development of the pupal female-flight muscles (and thus with significant cell division and differentiation), the VitellogeninA1 gene expresses in a tissue (adult fat body) which has already developed and whose cells are fully differentiated.

With expression of our previously validated lethal effectors in the fat body having no apparent phenotype, we chose to employ a non-cell-autonomous approach (Fig 1C) in order to maintain blood-meal inducible timing but target potentially more sensitive tissues. Widespread use against adult mosquitoes of insecticides that target voltage-gated sodium channels (VGSC) [26] at neuromuscular junctions indicated that this could be an appropriate target for an effector at this life-stage. We designed a synthetic, neurotoxic effector construct consisting of a fusion between the invertebrate-specific, VGSC-targeting, scorpion-toxin gene AaHIT from *Androctonus australis hector* [27, 28] and the secretory signal peptide from *Autographa californica* baculovirus major envelope glycoprotein (gp67) [29], under the transcriptional control of tetO. Our hypothesis was that, when combined with the VgA1-tTAV line, this construct would allow blood-meal inducible, fat-body specific expression of AaHIT, which would be secreted into the haemolymph after translation; a common feature of fat-body expressed proteins such as VitellogeninA1 [30], where it would circulate, eventually being made available to VGSCs at neuromuscular junctions. To test this we generated a mosquito line carrying tetO-gp67.AaHIT (tetO-AaHIT) and crossed it to the VgA1-tTAV line to look for a blood-meal inducible phenotype in the progeny under full factorial conditions (all three genotypes i.e. tetO-AaHIT, VgA1-tTAV and tetO-AaHIT+ VgA1-tTAV (VgA1>AaHIT), on and off-tetracycline).

**Fig 2:**
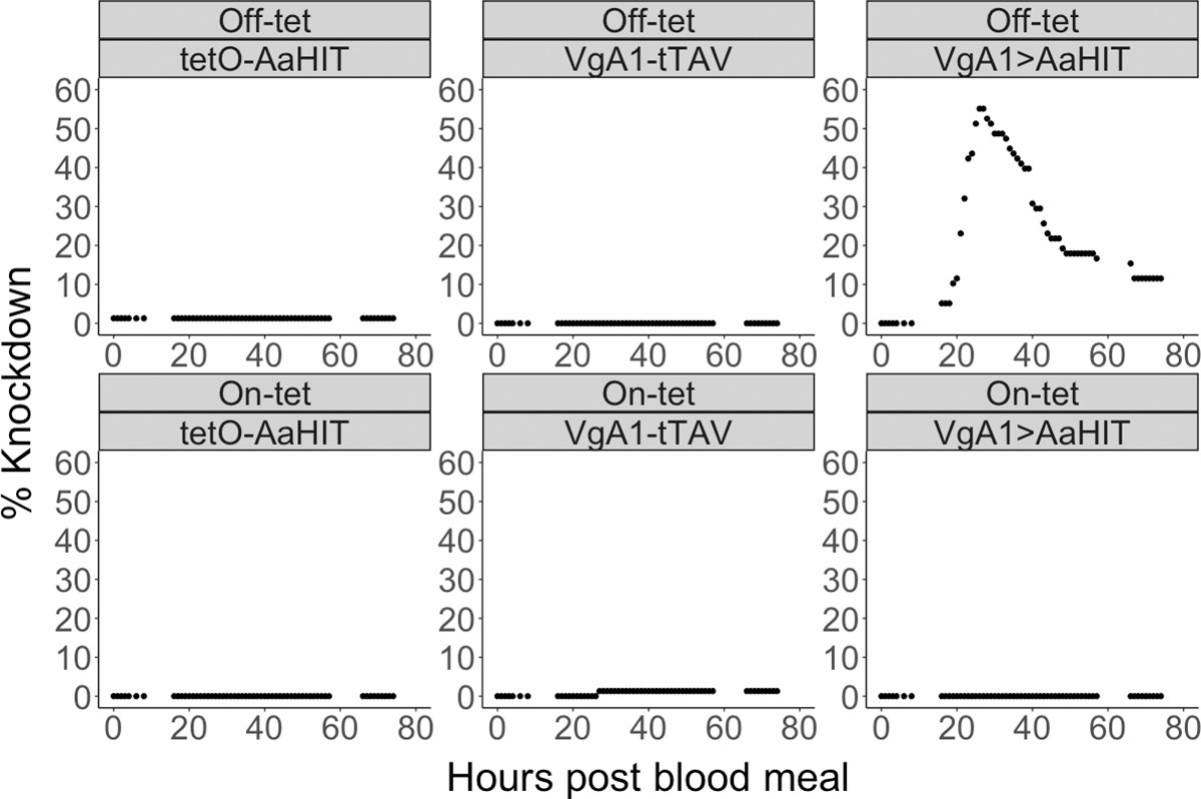
Repressible female knockdown phenotype in VgA1>AaHIT. Graphs showing the percentage of females in a cage showing knockdown phenotype over time. Each panel represents a cage with the tet-status (off-tet or on-tet) and genotype (VgA1-tTAV, tetO-AaHIT and VgA1>AaHIT) of females within that cage given in headers above each graph. Starting numbers of females in each cage were VgA1-tTAV _off-tet_ (n = 104), tetO-AaHIT _off-tet_ (n = 78), VgA1>AaHIT _off-tet_ (n = 78), VgA1-tTAV _on-tet_(n = 76), tetO-AaHIT _on-tet_ (n = 87) and VgA1>AaHIT _on-tet_ (n = 88). Y-axis gives the net number (knocked down–recovered) of females in each cage which were knocked down at any given time point pbm (x-axis). In all cages, females had ceased to knockdown prior to any individuals recovering and as such the peak of each graph represents the total number knocked down in that cage. Knockdown response was largely isolated to the VgA1>tTAV _off-tet_ cage in a temporal pattern in keeping with known expression behaviour of the *Aedes aegypti* VitellogeninA1 gene. A large percentage of paralysed females (77%) recovered from this knockdown effect and went on to lay eggs (analysed in S1 Table).

Beginning at 16h pbm, VgA1>AaHIT females reared in the absence of tetracycline (“off-tet”) exhibited loss of motor control and paralysis consistent with the known mode of action of AaHIT, an excitatory neurotoxin (Fig 2). “Knockdown” comprised a gradient of behaviours from mild, where females could not fly but staggered haphazardly on the bottom of the cage, to severe, where they lay on their dorsal thorax with their legs and wings convulsing asynchronously (see S1–S5 Videos for recordings of individual knockdown paralysis phenotypes). The proportion of the post-blood-feeding cohort that showed this phenotype increased rapidly from c. 20h pbm, consistent with the temporal pattern of tTAV expression in the VgA1-tTAV line. The last knockdown event occurred at 26h pbm at which point 44/78 (56.4%) of VgA1>AaHIT_off-tet_ females displayed the phenotype. Starting from 27h pbm, knockdown females began to recover (defined as fully regaining the ability to fly–see methods and S3–S5 Videos). By 67h pbm all knockdown females had either recovered or died (77.3% recovery) with the average time spent knocked down before recovery being 20.4h ± 1.94. This recovery behaviour suggests that the relatively brief expression of tTAV driven by VgA1 was insufficient to cause more than a sub-lethal effect and/or the mode of action of AaHIT did not result in irreversible depolarisation of the nerve axons/terminals, similar to some type I pyrethroids [26]. One single female each from the VgA1-tTAV_on-tet_ and tetO-AaHIT_off-tet_ cages were recorded as non-moving (at 27h and 1h pbm, respectively). However, it was immediately established that these females had died, rather than become paralysed. Analysis of cages where at least one individual died did not identify significantly higher levels of mortality in the VgA1>AaHIT_off-tet_ cage (which uniquely displayed the knockdown phenotype) compared to other genotype x tet-status cages (Pairwise Fishers exact test, VgA1>AaHIT_off-tet_: tetO-AaHIT_off-tet_, p = 0.096; VgA1>AaHIT_off-tet_: VgA1-tTAV_on-tet_, p = 0.096; VgA1-tTAV_on-tet_: tetO-AaHIT_off-tet_, p = 1).

**Fig 3:**
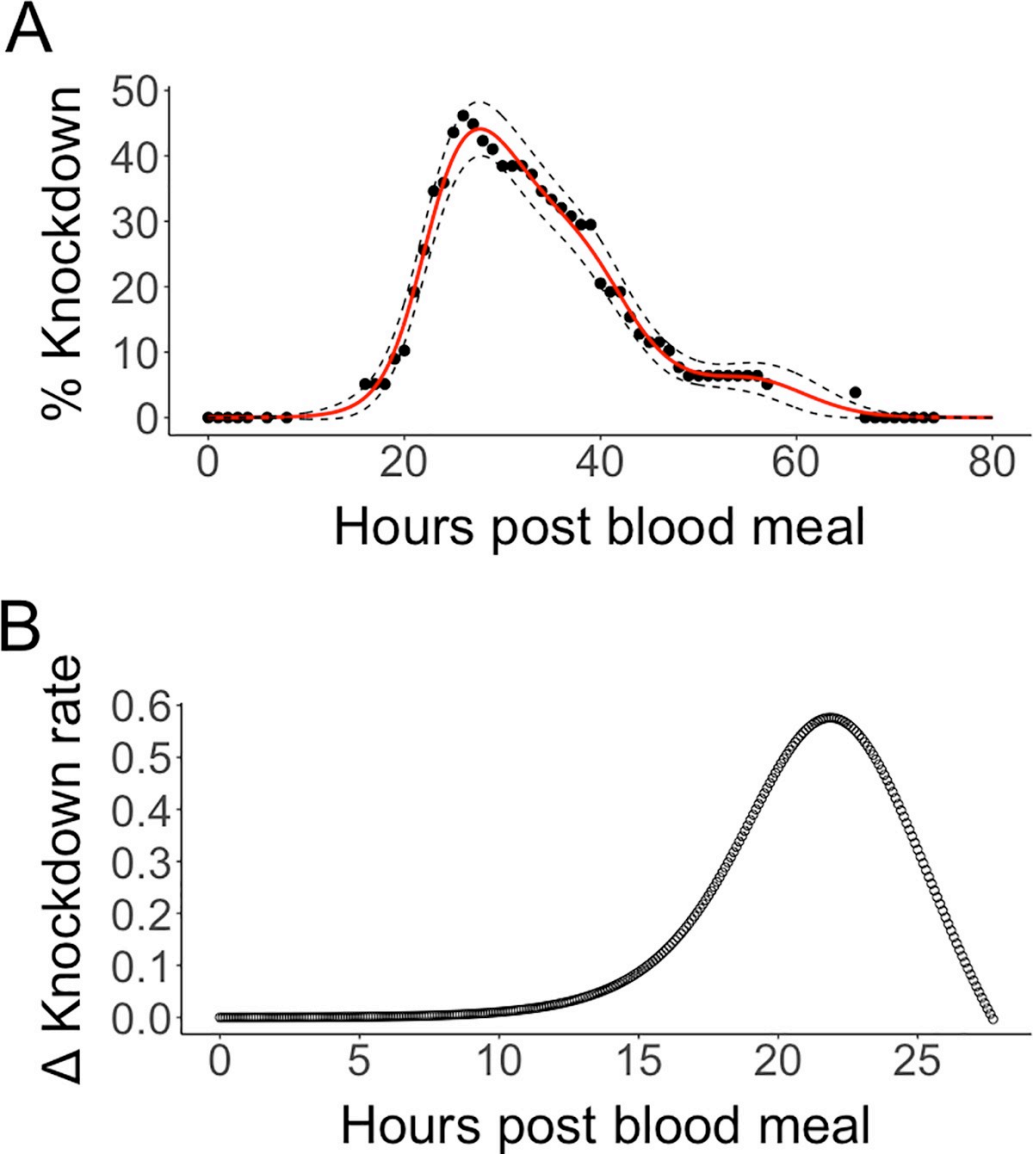
Generalized Additive Model of knockdown/recovery behaviour. Results of a binomial GAM model fitted to VgA1>AaHIT _off-tet_ cage knockdown data with non-recovered (dead) females removed prior to analysis (panel A). Raw data is represented by black dots with the red solid line showing the back-transformed output from the binomial GAM and black dashed lines showing the CI of the model. This model predicted the peak knockdown point at 27.7hrs pbm with 44.1% ± 2.11 of the cage paralysed. Output from the fitted GAM was used to assess the rate of change in knockdown phenotype over time (panel B). The period from 0–27.7 hrs pbm was divided into bins of 6mins. During each 6min bin the number of females showing the knockdown phenotype (as predicted by the GAM) was compared to that of the previous bin in order to calculate the rate of change during that time period. The X axis is limited to 27.7hrs as this was the maximum knockdown time predicted by the GAM. The bin where females were knocking down most rapidly was at 21.9hrs pbm during which time an estimated 0.576% of the cage began experiencing the knockdown phenotype.

As background levels of mortality were not significantly different than in control cages, data from females that died was removed prior to further analysis of the reversible knockdown phenotype in the VgA1>AaHIT_off-tet_ cage. A binomial Generalised Additive Model (GAM) was fitted to the data set (Fig 3A) using the mgcv R package and was found to have a highly significant smoothing term (X^2^ = 371, p = <0.005). This model predicted peak knockdown of the VgA1>AaHIT_off-tet_ cohort at 27.7h pbm with 44.1% ± 2.11 of the cage paralysed at this point. Using the output from this model, the rate of change in proportion knockdown over time was calculated (Fig 3B) and it was found that females in this cage were knocking down most rapidly at 21.9h pbm at an estimated rate of approx. 0.1%/min.

Surviving females were subdivided depending on whether they had experienced knockdown in the previous experiment (KnockDown = KD; this only applies to VgA1>AaHIT_off-tet_ females as no knockdown was observed for other genotypes or for VgA1>AaHIT_on-tet_) or had not (Not KnockDown = NKD). A Dunn test for multiple comparisons with Benjamini-Hochberg correction found no significant difference in the numbers of eggs laid by VgA1>AaHIToff-tet—KD females versus females from the same cage which had not knocked down (VgA1>AaHIT_off-tet_ -NKD; Z = 0.797, p = 0.213) suggesting no significant effect of paralysis on oviposition capability. VgA1>AaHIT_on-tet_ -NKD females laid significantly fewer eggs than the single hemizygotes, but did not differ significantly when compared to VgA1>AaHIT_off-tet_ irrespective of knockdown (S1 Table). A Poisson GLM found that the amount of time individual females spent knocked down did not significantly correlate with their subsequent fecundity (β = 0.498, t = -1.75, p = 0.091).

Although the knockdown phenotype was pronounced in those females experiencing it, the penetrance of this phenotype was incomplete. With VGSC resistance mechanisms such as the *kdr* mutation being extremely widespread, we were interested to examine whether the lack of sensitivity to the expressed scorpion toxin may also have a genetic basis. As a first step towards this we performed a second knockdown experiment using those females which had survived the first, immediately after oviposition. The aim of this experiment was to assess whether previous susceptibility to AaHIT knockdown predicted behaviour following a second exposure to this neurotoxin. As for the first blood meal, VgA1>AaHIT_off-tet_ females showed a knockdown response after a second blood meal (S4 Fig). However, unlike for the first blood meal, a small proportion of VgA1>AaHIT_on-tet_ females (2/30 = 6.66%) also showed a knockdown phenotype, albeit at a later time period (c. 10h later) than those fed off-tetracycline. Within the VgA1>AaHIT_off-tet_ females, a higher percentage of KD females became paralysed (8/38 = 21.0%) compared with NKD females (4/42 = 9.52%) although this effect was non-significant (binomial GLM: z = 1.41, p = 0.159). Therefore our data do not support the conclusion that there is a consistent susceptibility of individuals to knockdown that may have implied an underlying genetic basis for the lack of penetrance observed. However, to fully elucidate this, multi-generational heritability experiments would be required.

In general, levels of knockdown in this second experiment were more modest than in the first. One possibility for this may have been the reduced activity of the VgA1 promoter fragment. With VitellogeninA1 protein already having been produced for the first gonotrophic cycle, the levels of induction may be diminished on subsequent blood-feeding events leading to lower levels of tTAV/AaHIT expression from the integrated transgene. Alternatively, it is possible that sensitivity of the target neuromuscular junctions to AaHIT may exhibit plasticity with repeated sub-lethal exposure resulting in reduced response. Recovery behaviour was observed in this second knockdown experiment but this too was reduced compared to that seen previously (VgA1>AaHIT_off-tet_—KD = 3/8 recovered—average time spent knocked down before recovery = 18.3h ± 1.67, VgA1>AaHIT_off-tet_—NKD = 1/4 recovered—time spent knocked down before recovery = 25h). However, all females that were knocked down a second time (including those which initially recovered) died prior to oviposition. Together these results suggest that with increasing age/gonotrophic cycles, females may become less sensitive to the neurotoxic effects of AaHIT, however, the consequences for those that do become paralysed are more serious, resulting in significant mortality. We note that knockdown behaviour after the second blood-meal would be less relevant in any eventual field use as transgenic females would likely be required to not survive to a second blood meal in order to reliably reduce disease transmission.

As none of the females that were knocked down a second time survived to lay eggs, only VgA1>AaHIT_off-tet_ -NKD and VgA1>AaHIToff-tet—KD females which had not been knocked down during the second blood meal could be compared during a second oviposition assay. In effect, this comparison analysed the longer-term consequences on fecundity of a single, initial, knockdown. Results of a Gaussian GLM of log(eggs laid) suggested no significant difference in oviposition ability between the two groups of females (t = 0.966, p = 0.338).

Here we demonstrate for the first time an ‘action at a distance’ phenotype in a globally important human disease vector, *Aedes aegypti*. Using a synthetic neurotoxic effector engineered to be secreted out of the tissue in which it is produced, we achieved a non-cell autonomous, blood-meal-inducible paralysis phenotype. As with some insecticides which also target VGSCs, this knockdown was transient with affected females recovering and displaying no significant reductions in terms of fecundity or survival. Under field conditions, however, such prolonged incapacitation would likely be fatal unless the mosquito is able to find and maintain a safe resting place before onset of incapacitation. The lack of full penetrance and lethality allowed us to investigate this phenotype over multiple gonotrophic cycles. However, in order to reduce disease transmission in the field a more reliable mortality rate after the first blood-meal would be necessary. This might be achievable simply by testing additional insertion lines, or by design modifications such as integration of a positive-feedback system such that, once induced by the VgA1 promoter, tTAV could then continue to induce its own (and therefore AaHIT) expression indefinitely. Choice of promoter and integration site may also prove useful in maximising the competitiveness of transgenic larvae through limiting expression of a chosen effector to the post-blood-meal female. This consideration is of particular importance to GPM strategies such as those described here which aim to limit any deleterious effect until after the density-dependent stage. Additionally, alternative arthropod-specific neuroactive effectors could be tested for increased penetrance, for example the spider [31] or sea anemone [32] toxins. A class of potentially highly potent but as yet unrealised neuroactive effectors are the endogenous insect neuropeptides [33]. These small secreted molecules act on neuronal cell-surface receptors and are responsible for regulating an extensive range of insect behaviours from reproduction and diapause/migration through to feeding and homeostasis/development. The ability to manipulate these behaviours in insects holds great promise for development of pest management tools [34, 35]. Other bloodmeal-inducible promoters are also available, for example, the blood-meal-inducible, mid-gut specific CarboxypeptidaseA promoter [36], with different timing and tissue specificity. The diversity and flexibility of these non-cell autonomous phenotypes suggests that they will be extremely useful in engineering more potent and effective forms of genetic pest management.

## Methods

### General mosquito husbandry

*Aedes aegypti* mosquitoes (Liverpool wild type strain) were reared in standard insectary conditions including 12:12 hour light:dark cycle, 70% (±10%) relative humidity, 26°C (±1°C) and constant air circulation. Larvae were fed TetraMin ornamental Fish Flakes (Tetra GmbH) using the following feeding regimen per larva: 0.08 mg on day 1; 0.48 mg on day 2; 0.48 mg on day 3; 0.32mg on days 5–10; 0.06 mg on day 12, or were fed ad libitum in excess for the characterisation of reversible paralysis phenotype. Sex of pupae was determined by identification of the genital lobe. Identification of transgene by fluorescent profile was also conducted at pupal stage using a Leica 165FC microscope, VgA1-tTAV: 3xP3-ECFP or tetO-AaHIT: HR5-IE1-DsRed. Adult mosquitoes were maintained in BioQuip cages with a 10% sucrose solution and/or a water-soaked cotton wool pad. Bloodfeeds were conducted using a Hemotek blood feeder with defibrinated horse blood (TCS Bioscience).

### Synthesis of plasmid constructs

The VgA1-tTAV tissue specific construct (*piggyBac*[Hr5IE1-AmCyan-VgA1-tTAV-SV40]) (Genbank accession number: MK795197) was made by modifying *piggyBac*[Hr5IE1-AmCyan-Carb-tTAV-SV40]) plasmid to change the Carb promoter region to the VitellogeninA-1 promoter region [37]

The effector constructs are *piggyBac*-based and contain the Hr5IE1-DsRed2 transformation marker, tetO repeats and an open frame for the effector. The tetO-Michelob_X effector construct is as previously described [16]. The tetO-Reaper construct contains the *Drosophila melanogaster* Reaper (Gene Id: 40015) CDS and the tetO-AaHIT construct contains the full length AaHIT CDS, which was synthesised by Geneart (Germany) (Genbank accession number: MK795198)

The tetO-Dsred2 reporter line is as previously described [16].

### Generation of transgenic lines

Transgenic lines were created using protocols previously described [6, 16, 38]. Details of injection results, line selection and insertion site characterisation [39, 40] are provided in Supporting Information (S2 Table, S3 Table and S4 Table).

### Molecular/phenotypic analysis of lines

#### Molecular characterisation of the VgA1-tTAV line

To determine the relative expression of tTAV transcripts before and after a blood meal, quantitative PCR (qPCR) was performed. RNA was extracted using Tri Reagent (Ambion, USA) treated with DNase I enzyme (Roche, UK) and quantified on a Pharmacia Biotech GeneQuant II RNA/DNA calculator. cDNA was generated from 0.5 μg of RNA with the RevertAid reverse transcription kit (Fermentas, Lithuania), using random hexamer primers. A 0.5 μg sample of this cDNA was then used to conduct Taqman qPCR using primers and probes specific to the tTAV coding sequence (primers: 5’CATGCCGACGCGCTAGA and 5’-GGTGAACATCTGCTCGAACTCGAAATC; Probe: 5’-Hex-CGGGATTCACCCCGCACGATAGC). The 18S ribosomal gene was used as an endogenous control; (primers: 5’- GTATTACGGCGCGAGAGGTG and 5’-GAAAACATCTTTGGCAAATGCTT, 18S probe: 6-Fam-TTCGTAGACCGTCGTAAGACTAACTAAAGCG). Each 25 μL reaction mix contained 12.5 μl Taqman Gene Expression Master Mix (Applied Biosystems, UK) combined with the two sets of primers to a final concentration of 0.9 μM each and the two probes to a final concentration of 24 μM each. qPCR reactions were performed on a Mx3005P thermal cycler (Stratagene, USA) with the following thermal cycler conditions: 50°C for 2 min; 95°C for 10 min; followed by forty cycles of 95°C for 15 seconds and 60°C for 30 seconds. Data were analysed using the MxPro-QPCR software (Stratagene) and R statistical package (R Foundation for Statistical Computing.

#### Characterisation of tissue-specific tTAV expression in VgA-tTAV line

To confirm that tTAV was expressed in a tissue-specific manner, hemizygous VgA1-tTAV males were crossed to hemizygous females of the tetO-DsRed2 reporter line. Male and female G1 progeny were screened for the expression of the DsRed2 fluorescent protein in the fat body of adults before and 48 hours after a blood meal to examine the sex and tissue-specificity, as well as bloodmeal inducibility of the tTAV expression.

### Initial tetO-michelob_x and tetO-Reaper^KR^ survival analysis

VgA1-tTAV hemizygous individuals were crossed to the hemizygous tetO-michelob_x and tetO-Reaper^KR^ effector strains. Half of the progeny were reared on-tetracycline (tet) conditions and the other half were reared off-tetracycline by hatching separately in 200 ml of filtered water. Three hours later, tet was added to the ‘on-tet’ half to a final concentration of 30μg ml^-1^. 1000 L1 larvae were aliquoted into trays (n = 3 per treatment), and reared at 1 larvae ml-1. In parallel, wild-type larvae were hatched and aliquoted into separate trays and reared at the same density with no tet, and males were kept for crosses. Larvae were fed powdered TetraMin Ornamental Fish Flakes (Tetra GmbH, Germany) according to the feeding regimen described.

Pupae were screened for transformation markers and male pupae were discarded. Female adults were blood fed three days after the last adult eclosed, and to enable synchronous blood feeding, sugar was removed from the females’ cages the day before. To prevent damage to engorged females, insects that had not taken a blood meal were removed from the cages and were excluded from the analysis.

On the same day, wild-type males were added to cages at a 0.5:1 male:female ratio. Dead females were counted and removed from cages daily until the end of the experiment, with particular attention paid to 20–28 hours after a blood meal, which corresponds to previously published reports on the VgA1 promoter’s peak activities.

Eggs were collected from the cages overnight. Females were blood fed for a total of three gonotrophic cycles; non-engorged females were separated from cages after each blood meal.

Kaplan Meier survival analysis was carried out using the ‘Rcmdrplugin.survival’ package in the statistical program ‘R for Mac OS X Version 1’, available publicly from http://cran.r-project.org.

### Characterisation of reversible paralysis phenotype

#### Experimental setup

The parental cross employed c. 400 tetO-AaHIT hemizygous females mated to c. 300 hemizygous VgA1-tTAV males. F_1_ oviposition papers were then vacuum-hatched to aid synchronicity of the resulting larvae. At the pupal screening stage, males were discarded and the females were sorted into 3 transgenic genotypes (tetO-AaHIT hemizygotes, VgA1-tTAV hemizygotes and VgA1>AaHIT transhemizygotes with non-transgenic individuals discarded). Each female genotype cohort was further divided into two equal groups to eclose in individual ‘off-tet’ or ‘on-tet’ condition cages–see below. Liverpool strain ‘wild-type’ males of similar age in days post eclosion (dpe) were introduced in all cages at approximately 1:1 ratio with transgenic females. As such, six treatments cages were set up.

#### Knockdown 1

When adults were 5–7 dpe, each cage was starved of sucrose solution and water for 24 hours and 6 hours respectively before provision of the experimental blood meal. On-tet blood meal included tetracycline, in the form of doxycycline hydrochloride (Sigma-Aldrich), dissolved in water then supplemented into the bloodmeal to result in a final concentration of 30μg/μl. Off-tet blood meal contained no added tetracycline. The blood meal was offered for 1 hour. Non-engorged adults (including males) were then vacuum-pootered from the cage and discarded. 10% sucrose solution was reinstated (on-tet cage sucrose solution contained tetracycline at a final concentration of 30μg/μl).

In summary, females in all 6 cages were observed every hour for 74 hours. Small breaks of non-observed periods took place between 8–16 and 57–66 hours as preliminary experiments suggested these times were not illustrative of the knockdown phenotype. During each observation point, each cage was tapped vigorously, any mosquito that fell and could not fly away when subsequently investigated with forceps was considered to be knocked down. These individuals were carefully picked up from the floor of the cage and placed within a single polypropylene *Drosophila* vial, the vial was then closed using a foam plug. If the individual mosquito recovered then it was transferred to another tube with fine mesh secured over the aperture and a small sucrose dampened cotton wool pad placed upon the mesh.

Following their knockdown, mosquitoes within vials were also observed each hour to record potential recovery behaviour. Each vial was agitated through tapping and the resulting behaviour recorded by quantifying into 5 stages– 1: Fully paralysed, typically lying on dorsal side, legs waving or jerking when stimulated. 2: Standing upright, can orientate to stand upright if knocked over but cannot fly. 3: Short, hopping flight (<90mm, vial height). Unable to sustain flight or reach top of vial. 4: Ability to sustain flight to top of vial. 5: No movement.

#### Oviposition assay 1

At the end of the experiment (74 hours post blood meal), stage 4 mosquitoes were considered to have recovered from knockdown. These were then transferred into another *Drosophila* vial, prepared as an oviposition vial, with damp cotton wool in the bottom overlaid with a piece of filter paper with the aperture closed as previously with mesh. All stage 4 mosquitoes were VgA1>tTAV _off-tet_ as this was the only genotype which showed knockdown behaviour. In addition, a minimum of 30 females from four other cohorts were prepared similarly (see Table 1 below for details).

**Table 1:** Number of females from each cohort cage set up in Drosophila vials to assess egg-laying. NKD = those individuals which did **N**ot **K**nock **D**own in experiment 1. KD = those individuals which did **K**nock **D**own in experiment 1.

	VgA1-tTAV _off-tet_-NKD	tetO-AaHIT _off-tet_-NKD	VgA1>tTAV _off-tet_-KD	VgA1>AaHIT _off-tet_-NKD	VgA1>AaHIT _on-tet_-NKD
**No. females set up**	30	30	38	42	30

After 42 hours (2 night cycles) in the vials, the surviving mosquitoes were released into 5 cohort cages. Eggs from each vial were counted. Vials where no eggs were laid or had hatched were recorded but not included in further analysis.

#### Knockdown 2

A second knockdown experiment was performed on mosquitoes which had survived oviposition assay 1 to assess the degree to which individual behaviour in knockdown experiment 1 predicted behaviour during a subsequent blood meal. Each of the 5 cohort cages was starved and fed a blood meal as described previously (blood meal 2 took place 7 days after their first blood meal). Tetracycline status of the blood meal was maintained as per knockdown 1. Cages were observed as previously described with knockdown mosquitoes treated the same way. After 42 hours both knockdown and recovery had ceased and so observations were stopped.

#### Oviposition assay 2

Vial preparation as per oviposition assay 1. However, as none of the mosquitoes which knocked down in knockdown 2 recovered (all died), only mosquitoes from two transhemizygous cohorts (VgA1>AaHIT _off-tet_ -KD and VgA1>AaHIT _off-tet_ -NKD) were set up and compared in the second oviposition assay.

### Statistical analysis details

Details of individual statistical analysis are described in the text. All statistical analysis was conducted using R. In general, a GLM framework was preferred for data analysis. Models fit was checked using various transformations/variance structures and QQ-plot and checked for over-dispersion. If assumptions could not be met, a non-parametric alternative was chosen. When multiple comparisons were made this was first tested using an appropriate omnibus test followed by post-hoc MultiComp testing.

## Supporting information

S1 Table Statistical output of fecundity comparison- first oviposition. Output of a Dunn test for multiple comparisons with Benjamini-Hochberg correction comparing the number of eggs laid between females from each of the cages in the post blood meal observation experiment. Individuals from the VgA1>AaHIT _off-tet_ cage were separated into those which had shown the knockdown phenotype and those which had not (KD = KnockDown or NKD = Not KnockDown respectively).
(DOCX)

S2 Table Injection results for constructs VgA1-tTAV and tetO-AaHIT. (DOCX)

S3 Table Mendelian inheritance ratios of AaHIT Scorpion toxin transgene in five lines. *χ*^2^ was used to quantify the significance of the difference between observed transgenic (TG):non-transgenic (NTG) ratio and the expected 1:1 ratio according to Mendelian inheritance predictions, where *p*<0.05 represents a significant difference (*).
(DOCX)

S4 Table Genomic DNA sequences flanking transgene insertions. TTAA PiggyBac insertion site underlined. Genomic sequences flanking the transgene insertion sites were determined using protocols previously described (39), with slight modifications. Here, template genomic DNA was extracted using the DNeasy Blood and Tissue Kit (QIAGEN, Germany) and then digested using restriction enzymes *BamHI*, *Bg1II*, *DpnII*, and *MspI* (New England Biolabs, USA). Amplified fragments were visualised on an agarose gel and bands of the expected size were excised, purified (New England Biolabs), and sequenced (GATC Biotech, Konstanz, Germany). From the sequencing products a partial *piggyBac* sequence was identified and the 5’-end genomic flanking sequence of tetO-AaHIT and 3’-end genomic flanking sequence of VgA1-tTAV were determined. To confirm the other side of the genomic insertion of each line (3’-end genomic flanking sequence of tetO-AaHIT and the 5’-end genomic flanking sequence of VgA1-tTAV) primers were designed to flank the insertion site; one primer in the *piggybac* sequence and the other in the genomic sequence predicted to flank the insertion site. Amplified fragments were again visualised on an agarose gel and bands of the expected size were excised, purified (New England Biolabs), and sequenced (GATC Biotech). From the sequencing products a partial *piggyBac* sequence was identified and the predicted 3’-end genomic flanking sequence of tetO-AaHIT and 5’-end genomic flanking sequence of VgA1-tTAV were confirmed. Each genomic flanking sequence was compared to the *Aedes aegypti* L5 genome assembly (40) using the BLAST nucleotide analysis in VectorBase (41), and each produced only one full length hit of >99% sequence similarity. The 5’-and 3’-end flanking genomic sequences of the VgA1-tTAV line showed significant similarity to *Aedes aegypti* AAEL001357, and the 5’-and 3’-end flanking genomic sequences of the tetO-AaHIT line showed significant similarity to *Aedes aegypti* AAEL007505.
(DOCX)

S1 Fig Mean fold change of the tTAV transgene in VgA1-tTAV lines at different life stages. Expression levels were determined using Taqman based duplex real time qRT-PCR and are relative to expression in transgenic male pupae. The results were normalised against 18S RNA. Error bars show the standard error of the mean of the three experimental replicates, each performed on RNA extracted from three pooled individuals. Significant differences (p < 0.05) from expression from pre-blood meal adult females was analysed using one way ANOVA and are indicated by a *.
(TIF)

S2 Fig DsRed expression in VgA1>DsRed2 cross. VgA1-tTAV and tetO-DsRed2 lines were crossed and transhemizygous progeny reared in presence of (+) or absence of (-) tetracycline. Photo shows adult males and females (48h pbm) viewed under a DsRed fluorescence filter. High level induction of the DsRed reporter could be observed in the blood fed females with some low level expression in the males (concurring with previous tTAV qPCR assays). In both cases, rearing on tetracycline suppresses the expression of DsRed.
(TIF)

S3 Fig Confirmation of Michelob_X expression in 3 pooled adult 48hr blood fed females. tetO-Michelob_x line was crossed to either the VgA1-tTAV or to a WT background line. RT-PCR confirmed expression of Michelob_X and induction when crossed to the tTAV driver line. Visible expression in the absence of the driver construct represents low-level basal expression from the tetO-Michelob_X insertion site. Expected amplicon size = 332bp.
(TIF)

S4 Fig Knockdown response on second blood meal. Graphs showing the percentage of females in a cage showing knockdown phenotype over time. Each panel represents a cage with the tet-status (off-tet or on-tet), genotype (VgA1-tTAV, tetO-AaHIT and VgA1>AaHIT) and previous knockdown-status (knocked down in first blood meal = KD, did not knockdown in first blood meal = NKD) of females within that cage given in headers above each graph. Starting numbers of females in each cage were VgA1-tTAV _off-tet_ -NKD (n = 30), tetO-AaHIT _off-tet_ -NKD (n = 30), VgA1>AaHIT _off-tet_ -KD (n = 38), VgA1>AaHIT _off-tet_ -NKD (n = 42), and VgA1>AaHIT _on-tet_ -NKD (n = 30). Y-axis gives the net number (knocked down–recovered) of females in each cage which were knocked down at any given time point pbm (x-axis). In all cages, females had ceased to knockdown prior to any individuals recovering and as such the peak of each graph represents the total number knocked down in that cage. VgA1>AaHIT individuals showed a knockdown response. However, unlike the first blood meal experiment, this response was not restricted to those reared off-tet. No females which knocked down survived to egg-laying analysis.
(TIF)

S1 Video VgA1>AaHIT _off-tet_ group of females exhibits typical knockdown phenotype (stage 1) c. 20hrs post blood-meal: lying on dorsal side, unable to establish resting pose, legs waving, wings sometimes vibrating or twitching.
(MP4)

S2 Video VgA1>AaHIT _off-tet_ female within a drosophila tube exhibits typical knockdown phenotype (stage 1) c. 20hrs post blood-meal: lying on dorsal side, unable to establish resting pose, legs waving, wings sometimes vibrating or twitching.
(MP4)

S3 Video VgA1>AaHIT _off-tet_ female within a drosophila tube exhibits typical knockdown recovery phenotype (stage 2) post blood-meal: Often standing upright, can orientate to stand upright if knocked over but does not fly. (MP4)

S4 Video VgA1>AaHIT _off-tet_ female within a drosophila tube exhibits typical knockdown recovery phenotype (stage 3): Short hopping flights (<90mm, vial height), unable to sustain flight or reach top of vial. (MP4)

S5 Video VgA1>AaHIT _off-tet_ female within a drosophila tube exhibits typical knockdown recovery phenotype (stage 4): Ability to sustain flight to top of vial. At this stage, females were deemed to have ‘recovered’ for the purposes of analysis.
(MP4)
