# Supplementary material for: Engineered action at a distance: Blood-meal-inducible paralysis in Aedes aegypti
Source: PLoS Negl Trop Dis. 2019 Sep 3;13(9):e0007579. doi: 10.1371/journal.pntd.0007579 (PMC6719823; doi:10.1371/journal.pntd.0007579)
Supplement: S1 Table — Output of a Dunn test for multiple comparisons with Benjamini-Hochberg correction comparing the number of eggs laid between females from each of the cages in the post blood meal observation experiment. Individuals from the VgA1>AaHIT off-tet cage were separated into those which had shown the knockdown phenotype and those which had not (KD = KnockDown or NKD = Not KnockDown respectively). (DOCX) [file pntd.0007579.s001.docx]

|  | tetO-AaHIT _off-tet_ -NKD | VgA1>AaHIT _off-tet_ -NKD | VgA1>AaHIT _on-tet_ -NKD | VgA1>AaHIT _off-tet_ -KD |
| --- | --- | --- | --- | --- |
| VgA1>AaHIT _off-tet_ -NKD | Z= 2.98, p= 0.007* |  |  |  |
| VgA1>AaHIT _on-tet_ -NKD | Z= 3.88, p= 0.0005* | Z = 1.20, p = 0.165 |  |  |
| VgA1>AaHIT _off-tet_ -KD | Z = 2.20, p = 0.035* | Z = -0.797, p = 0.21 | Z = -1.91, p = 0.056 |  |
| VgA1-tTAV _off-tet_ -NKD | Z = 1.11, p = 0.165 | Z=-1.77, p = 0.063 | Z = -2.76, p = 0.009* | Z = -1.01, p = 0.173 |
