# Supplementary material for: Engineered action at a distance: Blood-meal-inducible paralysis in Aedes aegypti
Source: PLoS Negl Trop Dis. 2019 Sep 3;13(9):e0007579. doi: 10.1371/journal.pntd.0007579 (PMC6719823; doi:10.1371/journal.pntd.0007579)
Supplement: S2 Table — (DOCX) [file pntd.0007579.s002.docx]

| **Construct** | **Eggs injected** | **G0 survivors** | **Lines isolated** |
| --- | --- | --- | --- |
| VgA1-tTAV | 1654 | 149 | 1 |
| tetO-AaHIT | 980 | 97 | 5 |
