# Supplementary material for: Engineered action at a distance: Blood-meal-inducible paralysis in Aedes aegypti
Source: PLoS Negl Trop Dis. 2019 Sep 3;13(9):e0007579. doi: 10.1371/journal.pntd.0007579 (PMC6719823; doi:10.1371/journal.pntd.0007579)
Supplement: S3 Table — χ2 was used to quantify the significance of the difference between observed transgenic (TG):non-transgenic (NTG) ratio and the expected 1:1 ratio according to Mendelian inheritance predictions, where p<0.05 represents a significant difference (*). (DOCX) [file pntd.0007579.s003.docx]

| Effector line | TG:NTG | ***χ*^2^**  (df=1) | p-value | Effect Size $\boldsymbol{\emptyset}$ |
| --- | --- | --- | --- | --- |
| AaHIT-B2 | 40:109 | 31.95 | <0.001***** | 0.463 |
| AaHIT-D5 | 135:148 | 0.6 | 0.742 |  |
| AaHIT-F1 | 120:129 | 0.33 | 0.85 |  |
| AaHIT-H2 | 85:107 | 2.52 | 0.284 |  |
| AaHIT-I6 | 73:180 | 42.25 | <0.001***** | 0.681 |
