# Supplementary material for: Engineered action at a distance: Blood-meal-inducible paralysis in Aedes aegypti
Source: PLoS Negl Trop Dis. 2019 Sep 3;13(9):e0007579. doi: 10.1371/journal.pntd.0007579 (PMC6719823; doi:10.1371/journal.pntd.0007579)
Supplement: S4 Table — TTAA PiggyBac insertion site underlined. Genomic sequences flanking the transgene insertion sites were determined using protocols previously described (39), with slight modifications. Here, template genomic DNA was extracted using the DNeasy Blood and Tissue Kit (QIAGEN, Germany) and then digested using restriction enzymes BamHI, Bg1II, DpnII, and MspI (New England Biolabs, USA). Amplified fragments were visualised on an agarose gel and bands of the expected size were excised, purified (New England Biolabs), and sequenced (GATC Biotech, Konstanz, Germany). From the sequencing products a partial piggyBac sequence was identified and the 5’-end genomic flanking sequence of tetO-AaHIT and 3’-end genomic flanking sequence of VgA1-tTAV were determined. To confirm the other side of the genomic insertion of each line (3’-end genomic flanking sequence of tetO-AaHIT and the 5’-end genomic flanking sequence of VgA1-tTAV) primers were designed to flank the insertion site; one primer in the piggybac sequence and the other in the genomic sequence predicted to flank the insertion site. Amplified fragments were again visualised on an agarose gel and bands of the expected size were excised, purified (New England Biolabs), and sequenced (GATC Biotech). From the sequencing products a partial piggyBac sequence was identified and the predicted 3’-end genomic flanking sequence of tetO-AaHIT and 5’-end genomic flanking sequence of VgA1-tTAV were confirmed. Each genomic flanking sequence was compared to the Aedes aegypti L5 genome assembly (40) using the BLAST nucleotide analysis in VectorBase (41), and each produced only one full length hit of >99% sequence similarity. The 5’-and 3’-end flanking genomic sequences of the VgA1-tTAV line showed significant similarity to Aedes aegypti AAEL001357, and the 5’-and 3’-end flanking genomic sequences of the tetO-AaHIT line showed significant similarity to Aedes aegypti AAEL007505. (DOCX) [file pntd.0007579.s004.docx]

| Construct | Insertion site | Position | Location |
| --- | --- | --- | --- |
| VgA1-tTAV | GAGGTCTTCCGTTAGTAATAGAGGCTTGCA**TTAA**ACAAAGCTCGCATAATTTGTGATCTCGCCC | 3:47935789 (3p34) | AAEL001357; intron 1 |
| tetO-AaHIT | TACCTCTATCACCTCGTTACCTGGTGAGCT**TTAA**TTGAAACATAAAAACGCTAACTGAGCCATG | 2:223999439 (centromere p) | AAEL007505;  intron 2 |
