# Supplementary figures and images for: Engineered action at a distance: Blood-meal-inducible paralysis in Aedes aegypti
Source: PLoS Negl Trop Dis. 2019 Sep 3;13(9):e0007579. doi: 10.1371/journal.pntd.0007579 (PMC6719823; doi:10.1371/journal.pntd.0007579)

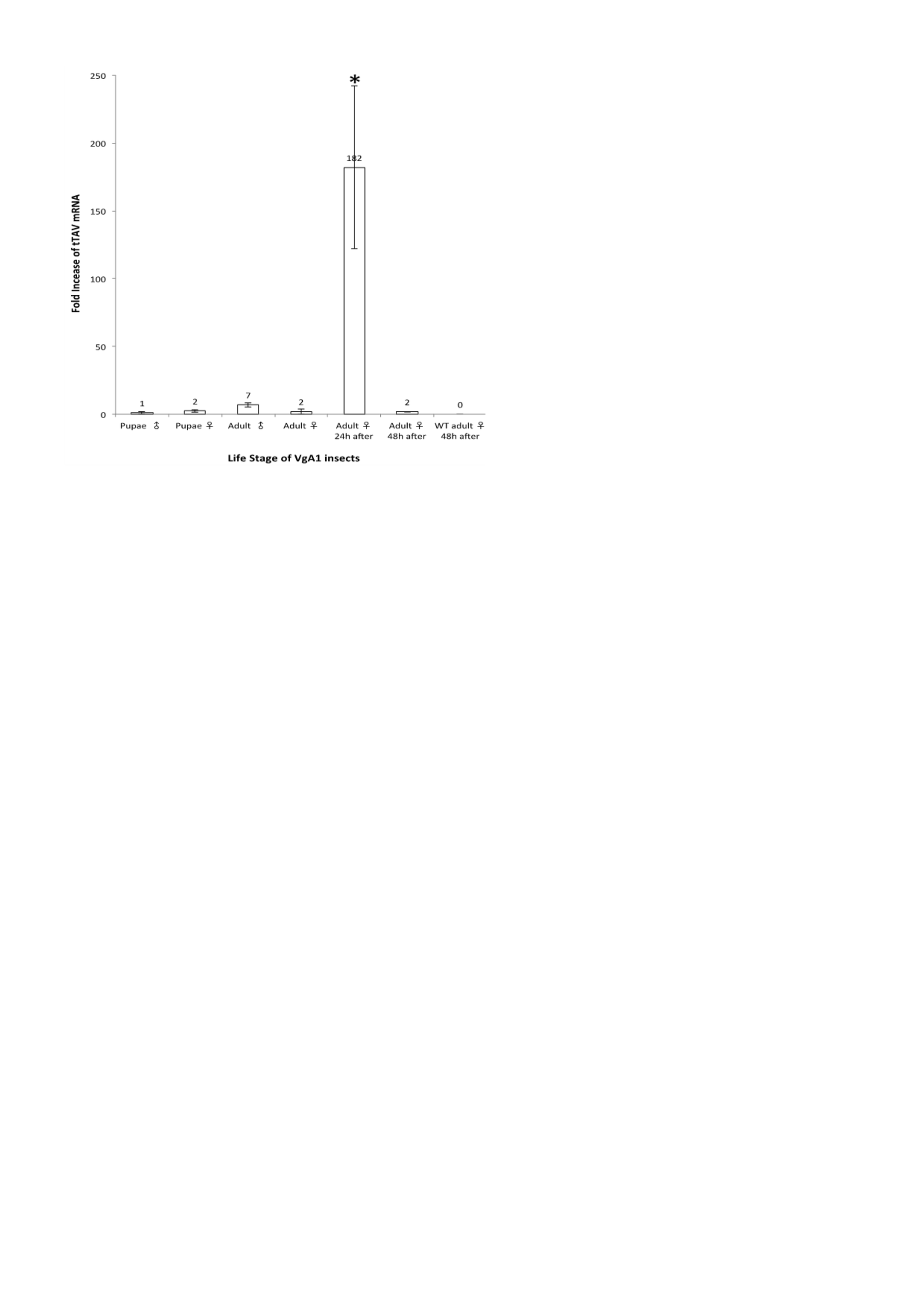

Supplement: S1 Fig — Expression levels were determined using Taqman based duplex real time qRT-PCR and are relative to expression in transgenic male pupae. The results were normalised against 18S RNA. Error bars show the standard error of the mean of the three experimental replicates, each performed on RNA extracted from three pooled individuals. Significant differences (p < 0.05) from expression from pre-blood meal adult females was analysed using one way ANOVA and are indicated by a *. (TIF) [file pntd.0007579.s005.tif]

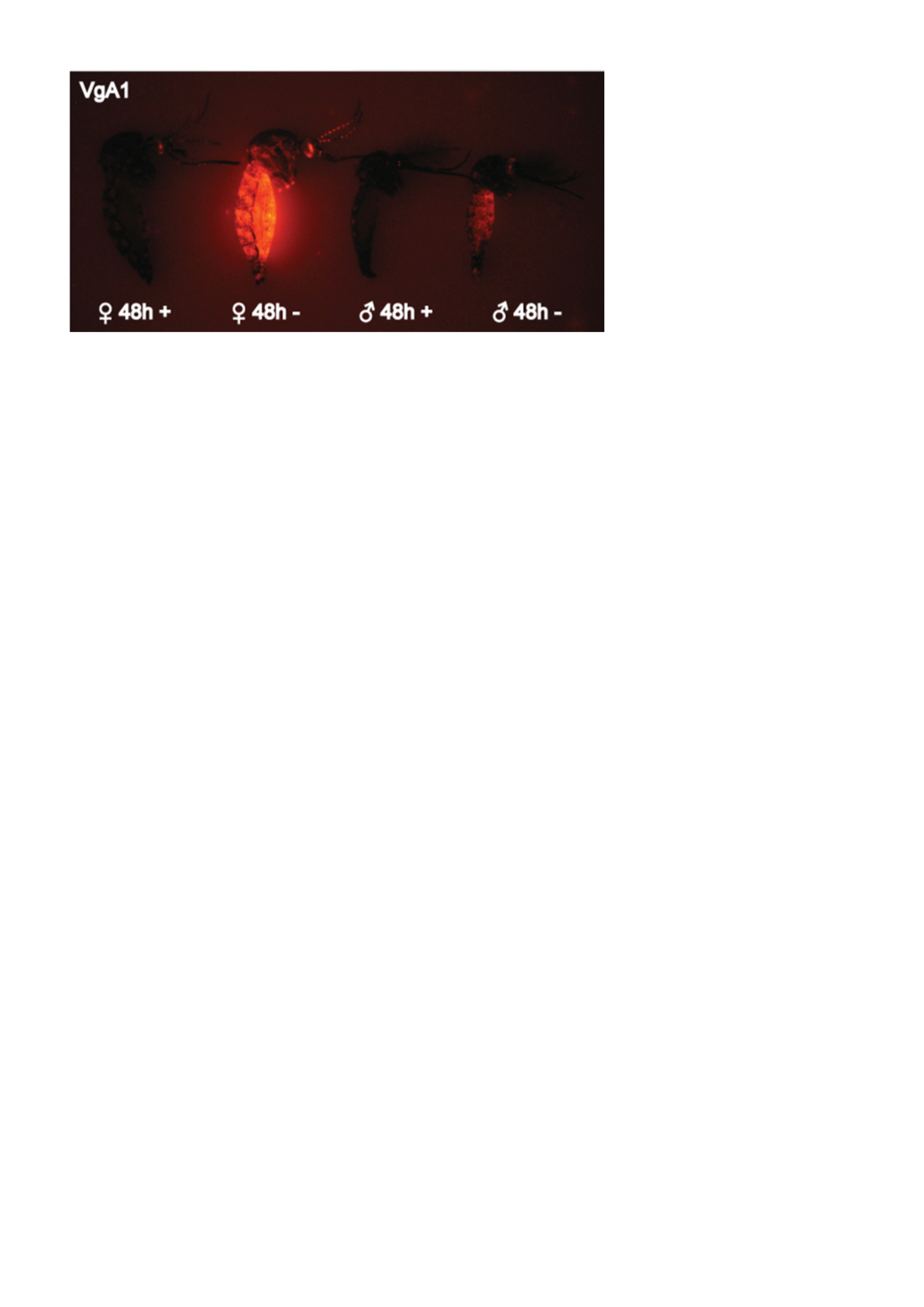

Supplement: S2 Fig — VgA1-tTAV and tetO-DsRed2 lines were crossed and transhemizygous progeny reared in presence of (+) or absence of (-) tetracycline. Photo shows adult males and females (48h pbm) viewed under a DsRed fluorescence filter. High level induction of the DsRed reporter could be observed in the blood fed females with some low level expression in the males (concurring with previous tTAV qPCR assays). In both cases, rearing on tetracycline suppresses the expression of DsRed. (TIF) [file pntd.0007579.s006.tif]

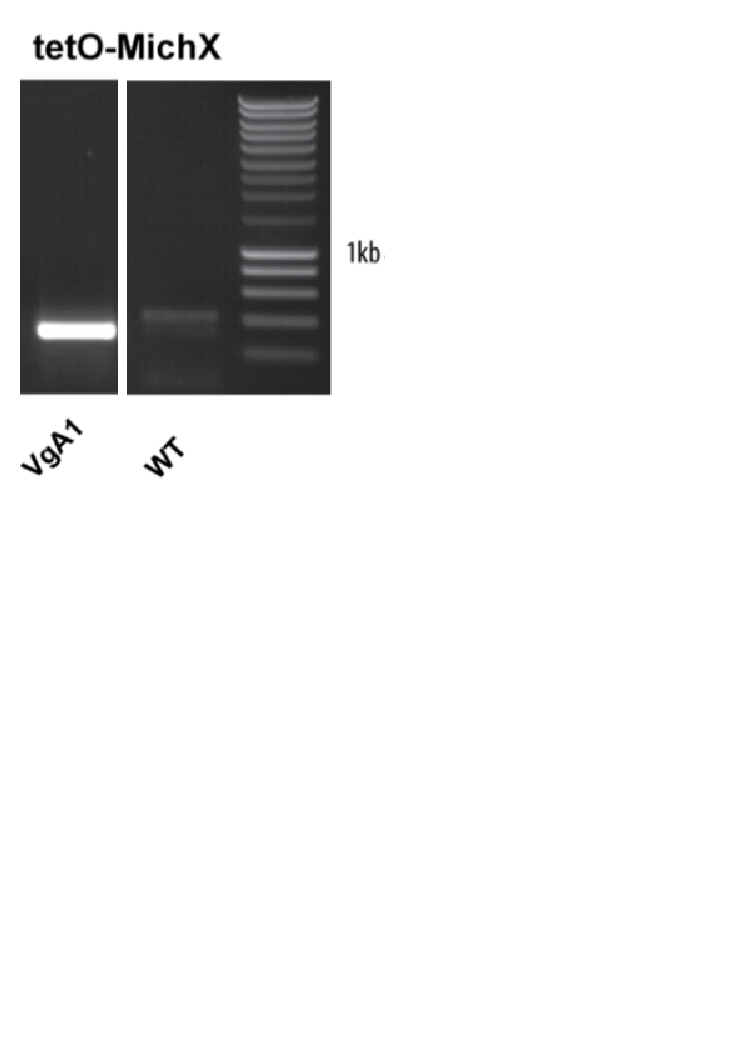

Supplement: S3 Fig — tetO-Michelob_x line was crossed to either the VgA1-tTAV or to a WT background line. RT-PCR confirmed expression of Michelob_X and induction when crossed to the tTAV driver line. Visible expression in the absence of the driver construct represents low-level basal expression from the tetO-Michelob_X insertion site. Expected amplicon size = 332bp. (TIF) [file pntd.0007579.s007.tif]

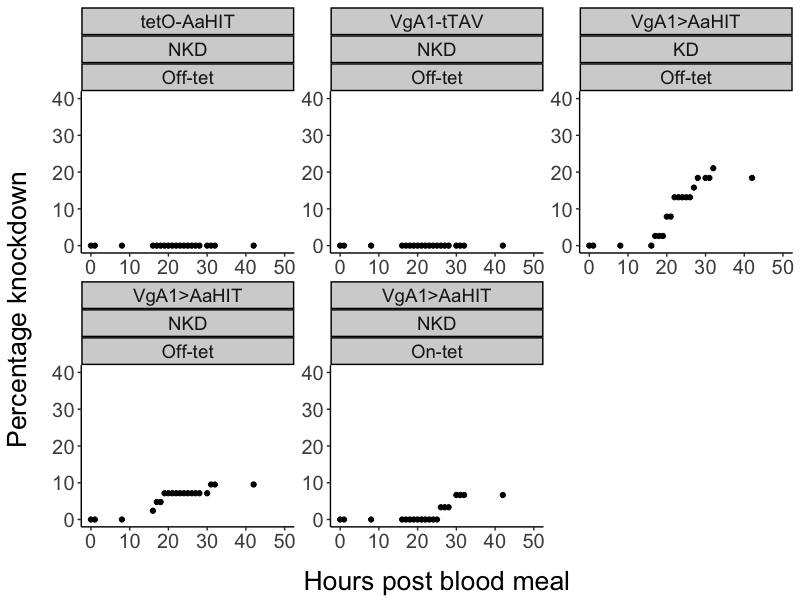

Supplement: S4 Fig — Graphs showing the percentage of females in a cage showing knockdown phenotype over time. Each panel represents a cage with the tet-status (off-tet or on-tet), genotype (VgA1-tTAV, tetO-AaHIT and VgA1>AaHIT) and previous knockdown-status (knocked down in first blood meal = KD, did not knockdown in first blood meal = NKD) of females within that cage given in headers above each graph. Starting numbers of females in each cage were VgA1-tTAV off-tet -NKD (n = 30), tetO-AaHIT off-tet -NKD (n = 30), VgA1>AaHIT off-tet -KD (n = 38), VgA1>AaHIT off-tet -NKD (n = 42), and VgA1>AaHIT on-tet -NKD (n = 30). Y-axis gives the net number (knocked down–recovered) of females in each cage which were knocked down at any given time point pbm (x-axis). In all cages, females had ceased to knockdown prior to any individuals recovering and as such the peak of each graph represents the total number knocked down in that cage. VgA1>AaHIT individuals showed a knockdown response. However, unlike the first blood meal experiment, this response was not restricted to those reared off-tet. No females which knocked down survived to egg-laying analysis. (TIF) [file pntd.0007579.s008.tif]
